# Efficacy of Young Cinnamomum zeylanicum Blume Bark on Hyperglycemia and PTPase Activity in Type 2 Diabetes

**DOI:** 10.7759/cureus.35023

**Published:** 2023-02-15

**Authors:** Anindita Mandal, Suresh K Sharma, Shashi Ranjan Mani Yadav, Anissa Atif Mirza, Mridula Singh Thakur, Sanjay Jachak, Sakshi Saini, Partha Roy, Ravi Kant, Meenaxi Patil

**Affiliations:** 1 Nursing, All India Institute of Medical Sciences, Rishikesh, IND; 2 Nursing, All India Institute of Medical Sciences, Jodhpur, IND; 3 Biochemistry, All India Institute of Medical Sciences, Rishikesh, IND; 4 Natural Product, National Institute of Pharmaceutical Education and Research, Mohali, IND; 5 Bioscience and Bioengineering, Indian Institute of Technology, Roorkee, IND; 6 Internal Medicine, All India Institute of Medical Sciences, Rishikesh, IND; 7 AYUSH, All India Institute of Medical Sciences, Rishikesh, IND

**Keywords:** ceylon cinnamon, diabetes mellitus, protein tyrosine phosphatase, fasting blood glucose, cinnamomum zeylanicum, plant compound

## Abstract

Diabetes is a major public health concern and natural easy-going remedies are being searched. Since *Cinnamomum zeylanicum *Blume has a low coumarin concentration and possible insulin-enhancing properties, it is preferred over all other cinnamon species. Although similar research has been done on humans, there have been very few studies on this particular species, and none among South Asians. Moreover, no human trial that properly described their intervening agent (*C. zeylanicum*) and checked its efficacy at the molecular level along with clinical variables was conducted. Therefore, the current research aimed to explore the effects of *C. zeylanicum *on the glycemic index, lipid profile, and expression of the protein tyrosine phosphatase 1 B (PTP1B) enzyme in the peripheral blood mononuclear cells (PBMC) in type 2 diabetes.

We examined the presence of bioactive compounds in young *C. zeylanicum* bark (Alba grade) from native Sri Lanka using gas chromatography-mass spectrometry, high-performance thin-layer chromatography, and thin-layer chromatography before introducing it in the clinical study where trans-Cinnamaldehyde was found to be a major chemical constituent (>60%). Then, from January 2020 to March 2022, a randomized double-blinded placebo-controlled trial was carried out in the Diabetic Clinic at AIIMS Rishikesh. A total of 154 diabetic patients were enrolled and were taken either cinnamon or placebo capsules (1.5 g/day) for 120 days on an empty stomach with warm water along with their conventional treatment. Reduction in fasting blood glucose levels in the cinnamon group was found -35.50% (95% CI, -173 to 58.4), whereas in the placebo group change was 5.00% (95% CI, -165 to 224). For glycosylated hemoglobin, it differed -0.85% (95% CI, -8.2 to 1.6) in the cinnamon group compared to the placebo where it was found 0.15% (95% CI, -6.1 to 5.5). PTP1B expression in PBMC was determined from pre- and post-trial blood samples using the Western Blot, and significant inhibition was also observed (p=0.039). The study result depicts, *C. zeylanicum* is emerging as a beneficial plant for type 2 diabetes in Northern India and could be used as an adjunctive treatment rather than as a standalone managerial remedy.

## Introduction

Type 2 diabetes mellitus (T2DM) is a chronic complex metabolic disease with impaired insulin sensitivity and diminished insulin secretion. It is caused by multiple etiologies and the confluence of genetic, environmental, and behavioral risk factors, including inactivity, sedentary behavior, cigarette smoking, and excessive alcohol consumption [1]. The prevalence of T2DM has been progressively growing globally. Among people over the age of 18 years, it has hiked from 4.7% in 1980 to 8.5% in 2014 and was expected to reach 9.3% in 2019 [2,3]. Limited options for decreasing blood sugar, adverse effects of modern medicine, unpleasant insulin therapy, unexpected hypoglycemia, and the expense of treatment necessitate the quest for a hassle-free cure. Since the 1990s, when nuclear receptor proteins that regulate gene expression were recognized as a potential therapeutic target against diabetes, cinnamon had become the subject of research [4]. The name "cinnamon" is derived from the Latin and medieval French intermediate forms of the word "kínnamon," which means "sweet wood" [5]. Various species belonging to the Lauraceae family are cultivated as sources of cinnamon spice such as "Ceylon cinnamon" or "*Cinnamomum zeylanicum*," "Chinese cinnamon," or "*Cinnamomum cassia*," "Vietnamese cinnamon" or "*Cinnamomum loureiroi*," "Malabar cinnamon" or "*Cinnamomum tamala*," "Indonesian cinnamon" or "*Cinnamomum burmannii*," and "Camphor laurel" or "*Cinnamomum camphora*" [6]. Cinnamon extracts (CE) containing trans-Cinnamaldehyde (majorly found in cinnamon bark) and phenolic compound (majorly found in leaf) may improve insulin sensitivity, and insulin release by inhibiting the enzyme protein tyrosine phosphatase 1B [6]. CE improves type 2 diabetes by prompting GLUT 4 translocation, a major glucose transporter in skeletal muscle and adipose tissue which plays a key role in the uptake of glucose from the bloodstream, storing it as glycogen, and oxidizing it to produce energy [7]. CE affects the genes related to lipid metabolism, e.g., fatty acid synthase, sterol-responsible binding protein-1c, lipoprotein lipase, hormone-sensitive lipase, etc., in a way to control the metabolic biohazards accompanied by diabetes [8]. Pyruvate kinase (PK) and phosphoenol pyruvate carboxykinase (PEPCK) are two more important enzymes that regulate hepatic glucose metabolism by increasing glycolysis and inhibiting gluconeogenesis. In diabetes, the diminished PK activity causes a reduction of glycolysis and elevated PEPCK activity catalyzes gluconeogenesis. CE reverses the increased hepatic PEPCK mRNA expression and restoration to near-normal values of PK [9]. It also decreases plasma oxidative stress markers and delays stomach emptying [10,11].

Though animal studies had provided evidence of the positive effects of cinnamon and there is gaining popularity in the traditional use against diabetes [12], very few human clinical trials on this particular species only in the Iranian population make up the paucity of sufficient scientific evidence as well as there has been no work proving it at the biochemical level in humans [13,14]. Studies on other species of cinnamon (*C. cassia *or *C. burmannii*) had also some clashes, e.g., lack of species name, origin, quality, purity, grade, etc. Methodological differences across previous studies (small sample size, dose, duration of trial, form of intervention, e.g., grind bark to water or alcohol extract), disparities in outcomes, and lack of hands-on proof from bimolecular levels in humans have made it imperative to carry out. Using cinnamon powder as a supplement in the diet could be inexpensive and combined with other diabetes care strategies it could lower blood sugar levels more successfully in nations like India where it is widely accessible. So, a well-defined methodology was used to develop and design the current randomized clinical trial (RCT) in North India.

## Materials and methods

For our research certified young *C. zeylanicum *Blume bark (Alba grade) was imported from the farm “True Ceylon Spice” of native Sri Lanka. After that, phytochemical analysis had been conducted using gas chromatography-mass spectrometry (GCMS), high-performance thin-layer chromatography (HPTLC), and thin-layer chromatography (TLC) in the Department of Natural products, National Institute of Pharmaceutical Education and Research (NIPER), Mohali, India. This was done to assess the purity and also to determine the active compound present in those imported barks prior to performing the trial and then crude bark powder was used for the actual randomized trial in the capsulated form.

Essential oil extraction

Dried bark (100 g powder) was subjected to steam distillation in a Clevenger-type apparatus for 8 hours. The distillate containing water along with essential oil was transferred into a 250 mL separating funnel. Dichloromethane (DCM) was added to the separating funnel for performing partitioning. The separating funnel was then capped tightly and shaken vigorously with occasional venting. When the two layers were separated, the lower DCM layer was transferred to a clean conical flask. The extraction process was repeated with two more portions of DCM and all of the DCM fractions were collected. Then, the pooled DCM layers were washed again with distilled water followed by separating into a dry container. The traces of aqueous solvent if present in the pooled fraction were removed by passing it through anhydrous sodium sulfate (Na_2_SO_4_). The combined DCM fraction was finally evaporated using a rotary evaporator. The residue was obtained in the form of a yellow oil (0.8 mL).

TLC analysis

**Figure 1:**
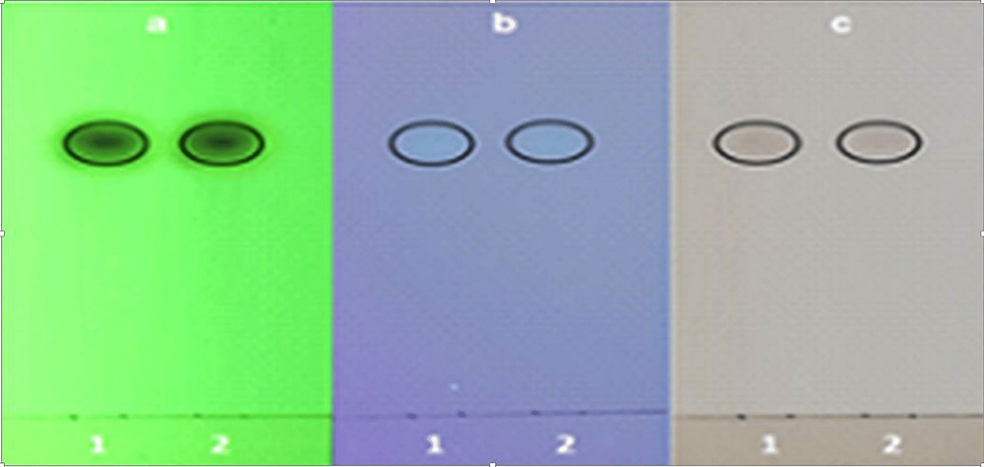
TLC profile of cinnamon oil with the trans-cinnamaldehyde standard. TLC profile at (a) 254 nm and at (b) 330 nm, (c) after derivatization (anisaldehyde sulphuric acid reagent) (1: cinnamaldehyde standard, 2: cinnamon oil).

The isolated compound (cinnamaldehyde) from *C. zeylanicum *bark oil was dissolved in chloroform (CHCl_3_) and then applied on a silica gel TLC plate and developed using a mobile phase comprising of toluene:ethyl acetate (EtOAc) (8:2). The TLC plate was visualized under UV light. The standard trans-cinnamaldehyde was used as a reference standard (Figure 1).

Soxhlet extraction

A 100 g of bark material was used for the soxhlet extraction using methanol (MeOH) solvent. After completion of the soxhlet extraction (24 hours) methanol was concentrated using a rotary evaporator to give 8.66 g of the dried extract. Of the total extract, 6 g was further used for partitioning using hexane, EtOAc, and n-butanol (n­-BuOH) in the order of increasing polarities. The amount of hexane extract, EtOAc extract and n-BuOH extract obtained after partitioning are 700 µL, 1.71 g, and 3.2 g, respectively.

HPTLC analysis

**Figure 2:**
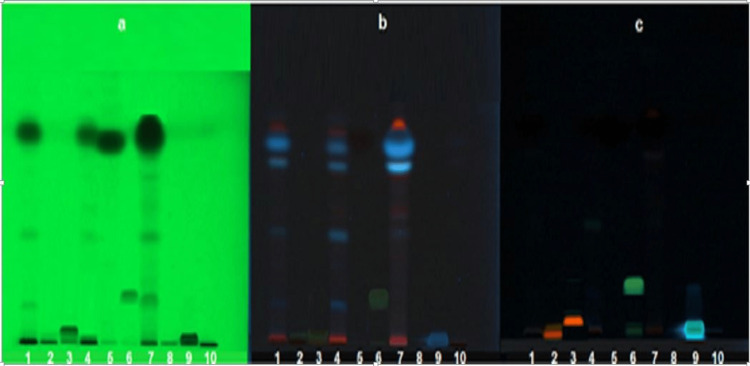
HPTLC profile at 254 nm (a), HPTLC profile at 330 nm (b), TLC profile after derivatizing with natural product (NP) reagent (c). 1: methanol extract, 2: rutin standard, 3: quercetin standard, 4: EtOAc extract, 5: cinnamaldehyde standard, 6: kaempferol standard, 7: hexane extract, 8: catechin standard, 9: caffeic acid standard, and 10: butanol extract. TLC Plate 1: Mobile Phase used: Toluene: EtOAc (8:2)

**Figure 3:**
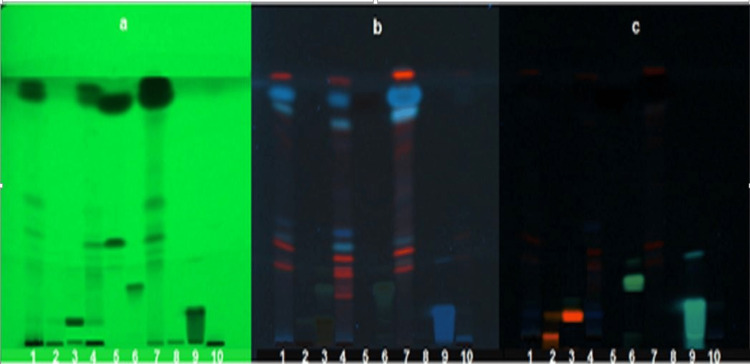
TLC profile at 254 nm (a), TLC profile at 330 nm (b), TLC profile after derivatizing with NP reagent (c). 1: methanol extract, 2: rutin standard, 3: quercetin standard, 4: EtOAc extract, 5: cinnamaldehyde standard, 6: kaempferol standard, 7: hexane extract, 8: catechin standard, 9: caffeic acid standard, and 10: butanol extract. TLC Plate 2: Mobile Phase: Chloroform: Methanol: Formic acid (9.5:0.4:0.1).

It was performed using two different mobile phases. The results with each of the mobile phases were recorded in Figures 2, 3.

HPTLC result

The TLC plates developed in two different mobile phases did not match the spots of standards which included rutin, quercetin, kaempferol, catechin, and caffeic acid. Only the presence of trans-cinnamaldehyde was confirmed with its standard using both HPTLC and TLC. The cinnamon oil percentage was calculated using the formula given as follows: % Oil = essential oil weight/sample weight × 100 × specific gravity = 0.8 mL/100g × 100 × 1.01 = 0.808. The percentage of cinnamon oil pre-reported in the bark of *C. zeylanicum *is 0.5-1%. The percentage of oil isolated was found to be 0.8% which is in the specified range [15].

GCMS analysis

**Figure 4:**
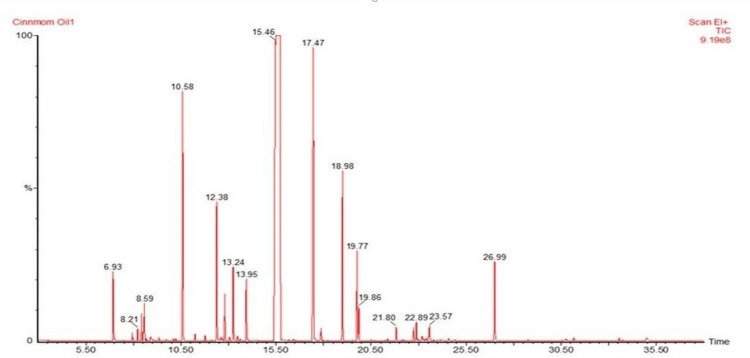
GC-MS spectrogram of cinnamon oil. GC-MS: gas chromatography-mass spectrometry

It was carried out to identify the chemical components present in the cinnamon oil obtained from the *C. zeylanicum* bark. Twenty-four chemical constituents were identified in the oil extracted from bark using the Clevenger apparatus viz. benzaldehyde, α-phellandrene, delta-3-carene, limonene, β-phellandrene, 3,7-dimethyl-1,6-octadien-3-ol, α-pinene, naphthalene-1,2,3,4,4a,7-hexahydro, endo-borneol, β-terpineol, d-limonene, cinnamaldehyde, eugenol, α-cubebene, copaene, α-cedrene, ylangene, caryophyllene, humulene, 3-methoxy cinnamaldehyde, caryophyllene alcohol, epiglobulol, α-santalol, and benzyl benzoate. The major components of the oil were found to be trans-cinnamaldehyde (33%) and its isomer (31%), followed by eugenol (7%), 3,7-dimethyl-1,6-octadien-3-ol (5%) caryophyllene (3%), naphthalene,1,2,3,4,4a,7-hexahydro (3%), benzyl benzoate (1.8%), d-limonene (1.5%), benzaldehyde (1.3%), and other constituents in trace amounts (Figure 4).

Trans-cinnamaldehyde is present as a major chemical constituent (>60%) in the cinnamon essential oil extracted through the Clevenger apparatus, confirmed by HPTLC, TLC, and GCMS analysis by using the reference standard of cinnamaldehyde. However, HPTLC analysis revealed the absence of rutin, catechin, kaempferol, quercetin, caffeic acid, and other polyphenols in the bark extracts of *C. zeylanicum*.

Capsule making

After the quality checking of cinnamon bark, the authors prepared both cinnamon and placebo capsules with the help of “Green Gold Pharmaceuticals,” Haridwar, India. These pharmaceuticals are a non-profit foundation for making Ayurvedic medicines, herbal extracts, essential oils, crude herbs, etc. A manual method was used and nearly about one-week period was taken to complete the whole process. Here, the cinnamon capsule contained 500 mg *C. zeylanicum *grind bark powder and the placebo capsule contained 500 mg grind Bengal gram flour. Both capsules were white in color and identical in shape. Both boxes were white, identical in shape, have the same aroma spray before closing, and were labeled as “Ayurvedic medicine for diabetes.” We didn’t make any extract capsules as our aim was to use whole-product in the clinical trial to evaluate effectiveness in their natural form.

Randomized controlled trial

The RCT was conducted from January 2020 to March 2022 in the Diabetic Clinic at All India Institute of Medical Sciences (AIIMS), Rishikesh. A total of 697 T2DM patients were assessed for the eligibility criteria and finally, 154 people were recruited as per the estimated sample size. The sample size estimation was done through GPower software 3.1 with moderate effect size 0.5, α 0.05, power 0.8, and 20% attrition rate. Patients with (1) non-insulin-dependent type 2 diabetes, (2) age more than 30 years (3) glycosylated hemoglobin 6.5, (4) fasting blood glucose level 126 mg/dL, (5) cooperative and want to participate, (6) no intake of herbs or other complementary therapy in recent eight weeks, (7) no acute infection (pneumonia, urinary tract infection, otitis), and (8) no insulin therapy were included in the study. Chain smoking, heavy alcohol, pregnancy and lactation, allergy or sensitivity to cinnamon, liver disease, and critically ill patients were excluded from the trial.

The study was randomized where block randomization had been done through a computer-generated random list created by sealedenvelope.com (block size four). Participants were randomized to both groups (77 persons in each group) and made their allocation concealed. The trial was double-blinded as researchers, e.g., recruiting physicians, blood sample collectors, and outcome assessors along with participants were blind. Allocation concealment and blinding were maintained throughout the trial. After taking written informed consent from participants and completing socio-demographic proforma, the pretrial blood sample was drawn for fasting blood glucose (FBG), glycosylated hemoglobin (HbA1c), and lipid profiles. Thereafter, cinnamon 500 mg three capsules daily were taken by the experimental group in the morning on an empty stomach with warm water for 120 days or four months. The control group did the same with placebo capsules (which look identical, white in color) containing finely grounded Bengal gram flour. The outcome was re-investigated at the end of the study as a routine checkup. Conventional management (dietary modification, exercise, and oral hypoglycemic agent, e.g., metformin, glimepiride, teneligliptin, was continued for both groups during the study. The third person who was a medicine box supplier and was responsible to analyze PTP1B was open and took 10% pre- and post-trial blood samples of the experimental group in a randomized way from the sample collection center separately.

Sample collection procedure

Whole blood sample in fasting (at least eight hours) condition was drawn from a brachial vein in a sitting position with the help of a needle 22-gauge, needle holder, and tourniquet from study participants at the sample collection center of AIIMS, Rishikesh. Blood samples were collected in one ethylenediaminetetraacetic acid (EDTA) (lavender color, 2 mL), one sodium fluoride (gray color, 2 mL), and one non-anticoagulant (red, 4 mL) vials for estimation of HbA1c, FBG, and lipid profile, respectively. Thereafter study participants were undergone with their concealed allocated intervention or placebo for four months with their previous prescribed treatment. After completion of the trial, fasting blood samples of FBG, HbA1c, and lipid profiles were re-collected in the same way. The collection of blood had been performed by health care professionals only. The aseptic technique was maintained. No extravasation injury was noticed.

Fasting blood glucose analysis

Blood glucose analysis was performed with the enzymatic hexokinase method on the Fully Automated Chemistry Analyser of Beckman Coulter Inc. (Brea, CA), model no. Au480, installed at the Clinical Biochemistry Laboratory, AIIMS, Rishikesh.

Estimation of glycosylated hemoglobin

HbA1c is the greatest indicator of average glucose levels during the previous one to three months. Estimation of HbA1c was conducted on TOSOH.HLC-723 (Tessenderlo, Belgium: Tosoh Europe N.V.) ion-exchange high-performance liquid chromatography principle at the Clinical Biochemistry laboratory AIIMS Rishikesh.

Lipid profile estimation

Fasting levels of serum triacylglycerol, total cholesterol, low-density lipoprotein, and high-density lipoprotein were also estimated on AU680 fully automated clinical chemistry analyzer at the Clinical Biochemistry Laboratory, AIIMS, Rishikesh.

Western blot

Expression of protein tyrosine phosphatase 1B was compared against beta-actin expression in the Bioengineering Lab of the Indian Institute of Technology, Roorkee. Total protein lysates were prepared from the collected patient PBMCs using radioimmunoassay (RIPA) buffer (50 mM Tris-HCL, 150 mM NaCl, 1% NP40, 0.1% sodium deoxycholate, 0.1% SDS, pH 7.4) containing protease inhibitor cocktail. Protein concentrations were determined using the Bradford method (BioRad) then equal amounts of proteins were prepared in Laemmli buffer for SDS-PAGE and separated by 12% SDS-polyacrylamide gel. The separated proteins were electro-transferred into a polyvinylidene difluoride (PVDF) membrane. The membranes were blocked with 3% BSA in TBST (20 mM, Tris, pH 7.5, 150 mM NaCl, and 0.1% Tween 20) for an hour at room temperature with constant rocking. The primary antibody (PTP1B rabbit the polyclonal antibody, catalog no. 11334-1-AP, Proteintech) was diluted at 1:2000 with 3% BSA in TBST and incubated for 2 hours at room temperature. Membranes were washed three times (10 minutes each) with TBST and incubated for 1 hour in horseradish peroxidase-conjugated secondary antibodies (Goat Anti-Rabbit IgG, catalog no. SA00001-2, Proteintech) diluted at 1:5000 in 3% BSA in TBST at room temperature [16,17].

The research has followed the guidelines of the Declaration of Helsinki and Tokyo for humans and was approved by the institutional review board (#AIIMS/IEC/18/577), AIIMS Rishikesh. The trial was registered in Clinical Trial Registry, registration no. CTI/2019/04/018386.

**Figure 5:**
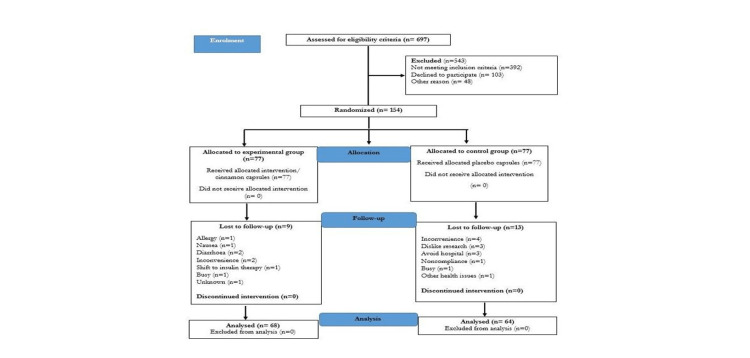
CONSORT flow diagram as per 2010 guidelines. CONSORT: Consolidated Standards of Reporting Trial

A total of 132 participants out of 154 had completed the trial. The reason for the 14% dropout was explained in Figure 5 (CONSORT flow diagram, as per 2010 guidelines). However, some of the reasons were allergies, nausea, diarrhea, inconvenience, non-compliance, avoiding the hospital, shift to insulin therapy, etc. No adverse effect was found during the trial and per protocol, analysis had been done for 68 participants in the experimental group and 64 participants in the control group.

Statistical analysis

Statistical analysis had been performed based on the data. Categorical data were reported as frequency and percentages. The difference between categorical data was assessed by chi-square test. Continuous data were reported as mean±standard deviation (SD), or median (minimum to maximum) for non-normally distributed data, which was tested by use of the Kolmogorov-Smirnov and Shapiro-Wilk test. The difference between the intergroup mean±SD was tested by an independent sample t-test and the inter-group median (minimum to maximum) was tested by the Mann-Whitney U test. The differences between clinical data (non-normally distributed) were analyzed by the Mann-Whitney U test (2-tailed non-parametric) where intra-individual delta values were taken for calculating the difference. P-values less than 0.05 were regarded as statistical significance. It was a per-protocol analysis, performed by the use of IBM SPSS Statistics for Windows, version 23 (Armonk, NY: IBM Corp.) [18].

## Results

Among socio-demographic details data regarding age, BMI, gender, marital status, education, occupation, religion, type of family, habitat, and duration of diabetes were obtained using a self-structured questionnaire. The data was tabulated and depicted in Table 1. Table 2 depicted the changes in fasting blood glucose, glycosylated hemoglobin, triglycerides, total cholesterol, low-density lipoprotein, and high-density lipoprotein from the initial to the end result of the trial.

**Table 1:** Socio-demographic characteristics of subjects from both groups. *P-value <0.05 is considered significant. **Independent sample t-test. ***Mann-Whitney U test. ****Chi-square test.

S. no.	Variable	Category	f (%)		p-Value*
			Experimental group (n=68)	Control group (n=64)	
1	Age	Mean±SD	52.09±11.88	53.55±10.78	0.463**
2	BMI	Median (min to max)	23.90 (18.9 to 32.4)	23.60 (17.6 to 36.9)	0.733***
2	Gender	Male	47 (69.1)	43 (67.2)	0.812****
		Female	21 (30.9)	21 (32.8)	
3	Marital status	Single	4 (5.9)	1 (1.6)	0.164****
		Married	63 (92.6)	59 (92.2)	
		Widow	1 (1.5)	4 (6.3)	
4	Educational status	Illiterate	2 (2.9)	7 (10.9)	0.170****
		Primary	6 (8.8)	9 (14.1)	
		Secondary	25 (36.8)	17 (26.6)	
		Diplomat and above	35 (51.5)	31 (48.4)	
5	Occupational status	Household	15 (22.1)	22 (34.4)	0.537****
		Service	28 (41.2)	17 (26.6)	
		Daily labor	4 (5.9)	3 (4.7)	
		Retired	10 (14.7)	10 (15.6)	
		Business	10 (14.7)	11 (17.2)	
		Unemployed	1 (1.5)	1 (1.6)	
6	Religion	Hindu	65 (95.6)	57 (89.1)	0.157****
		Muslim	3 (4.4)	7 (10.9)	
7	Type of family	Nuclear	31 (45.6)	34 (53.1)	0.387****
		Joint	37 (54.4)	30 (46.9)	
8	Habitat	Urban	54 (79.4)	47 (73.4)	0.418****
		Rural	14 (20.6)	17 (26.6)	
9	Duration of diabetes	<2 years	22 (32.4)	14 (21.9)	0.164****
		2-5 years	15 (22.1)	23 (35.9)	
		>5 years	31 (45.6)	27 (42.2)	

**Table 2:** Comparison of biochemical indices before and after trial. *P-value was calculated on intra-individual delta values and was considered as significant <0.05. Data represented as median (minimum to maximum); difference analyzed by Mann-Whitney U test.

S. no.	Variables	Experimental group (n=68)	Control group (n=64)	p-Value
1	FBG (mg/dL)			
	Initial	162.50 (109 to 370)	151.00 (102 to 335)	0.000*
	End	125.00 (80 to 292)	143.50 (83 to 383)	
	Difference	-35.50 (-173 to 58.4)	5.00 (-165 to 224)	
2	HbA1C (%)			
	Initial	8.95 (6.3 to 15.2)	7.80 (5.9 to 12.8)	0.000*
	End	7.55 (5.8 to 11.6)	7.80 (5.3 to 14.8)	
	Difference	-0.85 (-8.2to 1.6)	0.15 (-6.1 to 5.5)	
3	Triglycerides (mg/dL)			
	Initial	132.00 (48 to 453)	131.00 (48 to 574)	0.176
	End	123.80 (34 to 737)	143.00 (60 to 550)	
	Difference	-20.00 (-269 to 433)	-4.00 (-270 to 229)	
4	Total cholesterol (mg/dL)			
	Initial	196.00 (90 to 281)	188.00 (71 to 306)	0.74
	End	188.50 (80 to 319)	196.00 (75 to 306)	
	Difference	7.50 (-114 to 124)	4.00 (-78 to 153)	
5	LDL-C (mg/dL)			
	Initial	109.00 (40 to 168)	100.00 (30 to 203)	0.047^*^
	End	116.10 (28 to 185)	114.00 (37 to 195)	
	Difference	-4.00 (-83 to 89)	7.00 (-60 to108)	
6	HDL-C (mg/dL)			
	Initial	45.00 (25 to 84)	46.00 (33.2 to 184.8)	0.572
	End	46.00 (17 to 86)	46.00 (31 to 70)	
	Difference	-0.50 (-48 to 23.5)	-0.50 (-134.8 to 25.8)	

As indicated in Table 2, there was a significant decrease in FBG, and HbA1C levels in the cinnamon group compared to the placebo (p=0.000). No such reduction was observed in the placebo group. LDL-C level was found to increase in the placebo group compared to cinnamon (p=0.047). There were no notable changes in the levels of triglycerides, total cholesterol, and HDL cholesterol (p>0.05).

**Figure 6:**
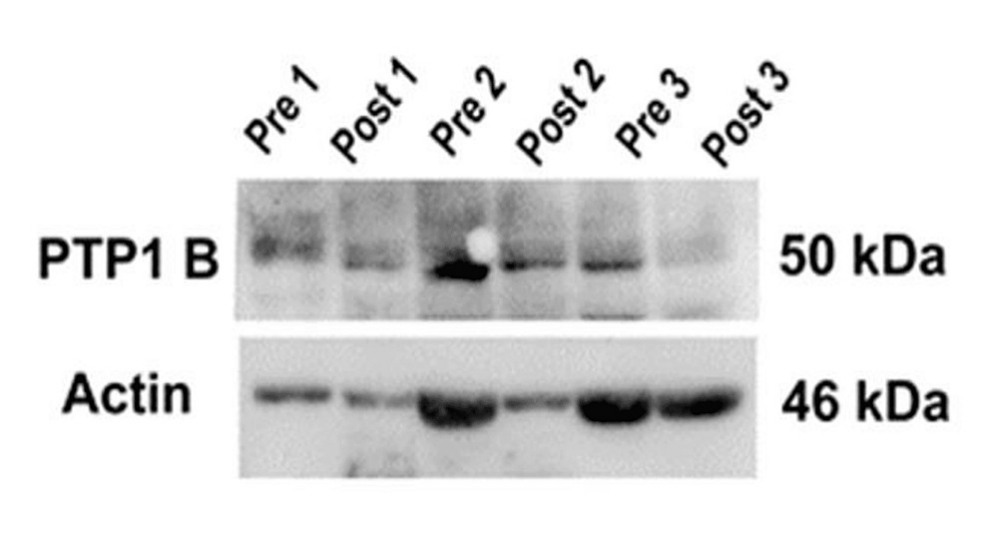
Changes in protein band of protein tyrosine phosphatase 1B and beta-actin before and after the trial.

To determine the inhibition of protein tyrosine phosphatase, the western blot method was applied and beta-actin was taken as a housekeeping gene. A total of 13 participants’ blood samples from the cinnamon group who had higher glycosylated hemoglobin at the time of the pre-test and maintained good compliance throughout the trial were collected randomly. One obese non-diabetic control was also taken. Therefore, every participant had four bands of protein: pre-PTP1B, post-PTP1B, pre-beta-actin, and post-beta-actin (Figure 6).

**Figure 7:**
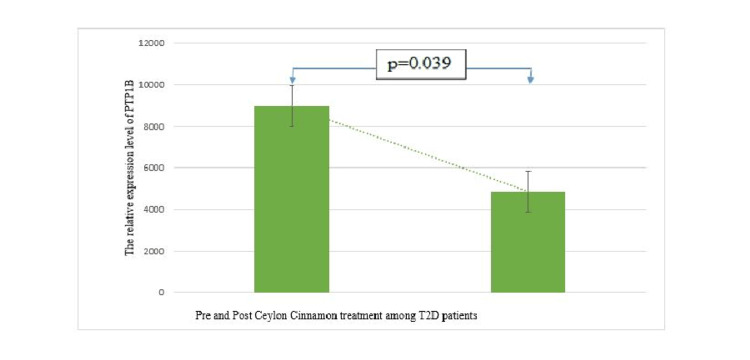
Expression of protein tyrosine phosphatase 1B in pre- and post-C. zeylanicum treatment.

Figure 7 represents the inhibition of protein tyrosine phosphatase in peripheral blood mononuclear cells of type 2 diabetes patients after consuming *C. zeylanicum* for four months. Student's t-test had been used for statistical analysis between the mean pre- and post-PTP1B band’s intra-optical density. Such results depict the anti-diabetic properties of *C. zeylanicum*.

## Discussion

Besides the multiple etiology, complex treatment regimens, unexpected hypoglycemia, high expenditure on treatment, patients' mistaken beliefs, and the negative impacts of modern medicine have all been cited as reasons for low compliance and poor adherence to current treatment methods for diabetes. While plant resources became a top priority in the quest for new medicines, cinnamon or dalchini, the common aromatic condiment obtained from the inner bark of the tree species genus Cinnamomum was touted as a promising spice for diabetes management [19].

A 62.09-89.31% of trans-Cinnamaldehyde could be found in cinnamon bark oil as a major compound and in the present research, it was found in nearly 64% which was in the pre-specified range [20-22]. Although the use of Ceylon cinnamon against diabetes was mentioned in the Charaka Samhita and cassia was well-known in Chinese folk medicine. Khan et al. in 2003 were the first to show scientific evidence through a human trial on cassia bark powder (1 g, 3 g, and 6 g) in Pakistan, where type 2 diabetic participants' fasting blood glucose and lipid profile was significantly reduced over the course of 40 days [23]. Following that trial, Crawford in 2009 in Las Vegas, Akilen et al. in 2010 in London, and Sengsuk et al. in 2015 in Thailand conducted research with a similar methodology and approved that 1-2 g of grind cinnamon cassia for 2-3 months could decrease HbA1C in type 2 diabetes [24-26].

But comparable results were not found by Vanschoonbeek et al. in 2006 in the Netherlands, Suppapitiporn et al. in 2006 in Thailand, Blevins et al. in 2007 in Oklahoma City, US, and Hasanzade et al. in 2013 in Iran [27-30]. The study of Vanschoonbeek et al. was primarily confined small sample size, a short duration, and solely female participants (post-menopausal women) who might be experiencing hormonal changes. In the study of Blevins et al., both groups were different by age, multi-ethnicity in a small study population was seen and the initial value of outcome variables was near to normal range. The study by Hasanzade et al. in 2013 majorly performed on a low socio-economic group of participants (housekeepers) which makes a methodological flaw in the research [30]. Besides those factors, the lack of a proper description of the intervening agent is a major discrepancy across all the studies. It's sometimes only described as to where it was acquired, e.g., from a local vendor. Cinnamon's origin, geographical wellness, and limit were unknown, and no validation or authentication was offered. It should be explained in detail, including the type of cinnamon, its origin, grade, whether it was new or old, and the optimal time to utilize it. Khan et al. in 2010 in Pakistan and Sharma et al. in 2012 in India didn't even specify the species' name though these studies conveyed a significant decrease in anthropometric, glycemic, and lipid index of patients with type 2 diabetes after consuming cinnamon bark [31,32].

When talking about extract, Ziegenfuss et al. in 2006 and Anderson et al. in 2016 employed cassia extract 500 mg/day for three and two months, respectively, and found reduced plasma glucose in type 2 diabetes [33,34]. The use of cassia extract for diabetes was positively impacted by intergroup differences in glycosylated hemoglobin along with fasting blood glucose levels by Lu et al. 2012 [35]. However, in those studies, there was no precise explanation of extract synthesis. Depending on the species and even the formulation, the number of active molecules (cinnamaldehyde otherwise polyphenols) may vary. Furthermore, phytochemicals may be harmed by differences in manufacturing techniques, because herbal drugs aren't subjected to similar quality control standards as other pharmacy items. Because cinnamon contains an evaporating agent, the essential oil might evaporate over time if it was old or mishandled, and there would be no active molecule to work with at that moment. The species of cinnamon was predetermined in the current investigation, along with the plant's age, location, and quality of the sticks. According to the plant list at http://www.theplantlist.org, *C. zeylanicum* Blume is a synonym of *Cinnamomum verum *J. Presl which is currently accessible in a variety of forms, including quills (usually 42-inch cinnamon sticks), quilling (broken cinnamon tubes), bark powder, bark oil, and leaf oil. The diameter of the cinnamon quills is divided into the following four classes in Sri Lankan grading: Alba (<0.24 inches), Continental (<0.63 inches), Mexican (<0.75 inches), and Hamburg (<1.3 inches). *C. zeylanicum *bark from native Sri Lanka was collected as young, fresh bark (Alba grade) for investigation. It was also measured whether the product was pure and legitimate and whether trans-cinnamaldehyde and eugenol, two active compounds, were present in the ground bark, and capsules were prepared just after grinding for best durability and to provide an equal quantity to the study subjects.

**Figure 8:**
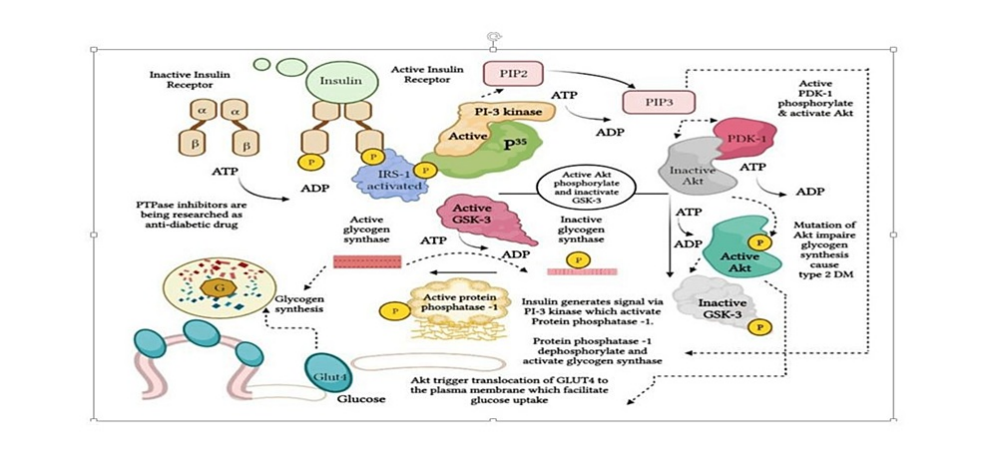
Schematic representation of active cinnamon compound in insulin signaling transduction. The image is created by the author (Anindita Mandal) of this study.

Vafa et al. carried out the first study on *C. zeylanicum *in Tehran, Iran, in 2012 where 44 participants with type 2 diabetes were enrolled (22 persons in each group; cinnamon and placebo), and 3g of grind *C. zeylanicum *was administered in the treatment group for eight weeks. Despite the study's small sample size and brief duration, the cinnamon group showed significantly lower FBG, HbA1c, and triglyceride levels than others [13]. Similar results regarding *C. zeylanicum *had also been found in the study by Zare et al. in 2019 in Iran [14]. The study was conducted on a relatively large sample size (138 participants) where cinnamon supplementation for three months significantly decreased levels of fasting and post-prandial blood glucose, HbA1c, triglycerides, total cholesterol, LDL cholesterol, and a significant increase in HDL cholesterol. The current research had been performed on 132 participants, and 1.5 g grind *C. zeylanicum *bark per day for four months resulted in a significant reduction in fasting blood sugar, and glycosylated hemoglobin among type 2 diabetic patients. Though present research didn’t observe any change in the levels of triglyceride and other lipids which might be related to a low dose of cinnamon or the near-normal value of the initial level. Along with significant inhibition of PTPase expression in peripheral blood mononuclear cells after four months of oral consumption of Ceylon cinnamon bark evidences it as an anti-diabetic herb from a biomolecular level (Figure 8).

When insulin binds to α unit of the insulin receptor, phosphorylation of tyrosine protein residue of β unit takes place. Kinase enzyme helps in the addition of phosphate from ATP to tyrosine amino acid is termed tyrosine kinase. The opposite enzyme protein tyrosine phosphatase removes a phosphate group from the target molecule and causes de-phosphorylation that inactivates insulin receptors followed by insulin resistance [36-38]. There is evidence that certain diabetic individuals have a variant of PTPase that is abnormally active and for this purpose, PTPase inhibitors, e.g., cinnamon are being explored (Figure 8) [39]. The result was similar to the study by Saifudin et al. in 2013 and Imparl-Radosevich et al. in 1998. Water and methanol extracts of *Cinnamomum burmannii* exhibited ≥70% inhibition at 25 μg/mL (IC50, 2.47 μg/mL) which was comparable with that of the positive control, RK-682 (IC50, 2.05 μg/mL). The PTP1B inhibitory activity of the constituents of *C. Burmannii* was then evaluated and trans-cinnamaldehyde (5; IC50, 57.6 μM) was found as an active constituent [40,41].

Though there's the fact that this type of human trial is difficult, as it would be among primary care patients. Trials of this nature could not be tightly controlled. Doctor shopping, medication changes, dose adjustments by patients exclusively, treatment discontinuation, participants' failure to comply with dietary restrictions, workouts, interactions with several pharmacotherapies, and lack of follow-up all had a significant impact on the trial's outcome. In the present study, researchers tried to control the extraneous variables by discussing with participants for both groups. The researchers didn’t explore about contents of the capsules, neither cinnamon nor placebo, and never prioritize anyone in front of the participants.

Major limitations of the present study are as follows: this is a single-centered study in single ethnic group and only one enzyme PTPase was studied through a single technique. Because topographical changes affect body mechanics, the study might be reproduced as a multi-centric trial including a broad population in a different geographic area. Instead of using the western blot, other biochemical tests could be utilized to acquire more specific information at the molecular level. Genes involved in the insulin pathway, rather than the insulin molecule, could also be assessed.

## Conclusions

*C. zeylanicum *has the potential to improve the glycemic index in persons with poorly managed diabetes. Its anti-diabetic properties have made it an herbal adjuvant treatment modality. This was further substantiated by the fact that ingestion of Ceylon cinnamon inhibited protein tyrosine phosphatase in peripheral blood mononuclear cells of type 2 diabetic individuals. Triglycerides, total cholesterol, and HDL-C levels were all unaltered. The clinical relevance and durability of these effects in multi-centric trials have yet to be determined. It's still worth looking into genes linked to glycemic pathways after consumption of *C. zeylanicum *as future prospects of research.
